# Breast cancer incidence subsequent to surgical reduction of the female breast.

**DOI:** 10.1038/bjc.1996.172

**Published:** 1996-04

**Authors:** M. Baasch, S. F. Nielsen, G. Engholm, K. Lund

**Affiliations:** Danish Cancer Society, Division for Cancer Epidemiology, Copenhagen.

## Abstract

The incidence of breast cancer among 1240 women who were treated surgically for breast hypertrophy in Copenhagen, Denmark between 1943 and 1971 was determined and compared with age- and calendar period-specific rates for the Danish female population. A total of 32 cases of breast cancer had developed by the end of 1990; the expected number was 52.55, yielding a relative risk (RR) of 0.61 [95% confidence interval (CI) 0.42-0.86]. The greatest reduction in risk was observed for women who had 600 g or more of breast tissue removed (RR=0.30; 95% CI 0.10-0.69). This suggests that the number of potential foci is important for cancer development in the female breast. In the group of women who were operated on before the age of 20, four cases of breast cancer developed, compared with 2.23 expected cases, to give an RR of 1.79, suggesting that the aetiology of their breast hypertrophy may be different from that for the rest of the group.


British Journal of Cancer (1996) 73, 961-963
© 1996 Stockton Press All rights reserved 0007-0920/96 $12.00

### Breast cancer incidence subsequent to surgical reduction of the female breast

M Baasch[1], SF Nielsen[2], G Engholm[1] and K Lund[3]

*[1]Danish Cancer Society, Division for Cancer Epidemiology, Strandboulevarden 49, Box 839, DK-2100 Copenhagen Ø; [2]Institute of Mathematical Statistics, University of Copenhagen, Universitets parken 5, DK-2100 Copenhagen Ø; [3]Professor Dr. Odont, Brohusvej 1, DK-7490 Aulum, Denmark.*

**Summary** The incidence of breast cancer among 1240 women who were treated surgically for breast hypertrophy in Copenhagen, Denmark between 1943 and 1971 was determined and compared with age- and calendar period-specific rates for the Danish female population. A total of 32 cases of breast cancer had developed by the end of 1990; the expected number was 52.55, yielding a relative risk (RR) of 0.61 [95% confidence interval (CI) 0.42-0.86]. The greatest reduction in risk was observed for women who had 600 g or more of breast tissue removed (RR=0.30; 95% CI 0.10-0.69). This suggests that the number of potential foci is important for cancer development in the female breast. In the group of women who were operated on before the age of 20, four cases of breast cancer developed, compared with 2.23 expected cases, to give an RR of 1.79, suggesting that the aetiology of their breast hypertrophy may be different from that for the rest of the group.

**Keywords:** breast cancer risk; breast hypertrophy; reduction mammoplasty

As women with breast hypertrophy may have their breasts surgically reduced, the natural question is whether this operation influences their risk for breast cancer. Animal experiments have suggested that the damage to the mammary ducts caused by mammoplasty leads to the evolution of stasis and hence to cancer (Fekete and Green, 1936). It has also been suggested, however, that surgical reduction of the breast decreases the incidence of breast cancer by reducing the number of potential foci for cancer development (Strömbeck, 1964; Rees and Coburn, 1972).

This study is a second follow-up of a cohort originally studied by Lund *et al.* (1987), comprising 1283 women operated on for breast hypertrophy between 1 January 1943 and 31 August 1971, who were followed up to 31 December 1982. The original study found a reduced incidence of breast cancer among these women in comparison with the Danish female population. The cohort has now been followed up to 31 December 1990.

### Materials and methods

The study group consisted of all women with breast hypertrophy treated by reduction mammoplasty at five surgical departments in Copenhagen, Denmark, between 1 January 1943 and 31 August 1971, and three patients operated on before 1 January 1943. The material was collected by examining diagnostic indices and lists of operations. The hospital records of 31 patients could not be located and these patients were excluded from the analysis. In the first study five patients were included twice in the study population, which therefore consisted of 1278 women. Six patients were lost to follow-up; in our follow-up, one patient was excluded as she appeared to have died before the operation. Thus, our study group consisted of 1240 patients. Information was obtained from hospital records on age, county, type of surgical technique and amount of removed tissue.

Several surgical techniques were used: the reduction was bilateral in 1201 cases, right-sided in 24 cases, and left-sided in 15 cases. The amount of tissue removed was unknown for 36 patients.

Correspondence: G Engholm
Received 21 September 1995; revised 23 October 1995; accepted 23 October 1995

On the basis of the patient's personal identification number or, if this was lacking, the patient's name and date of birth, the Central Population Register was consulted for date of death or emigration. Cases of breast cancer were identified by cross-linkage with the Danish Cancer Register. The period of follow-up was from the time of reduction mammoplasty to the first of the following events: time of breast cancer diagnosis, death, emigration or 31 December 1990. The three women operated on before 1943 were followed from 1 January 1943 because information on breast cancer incidence in the population was lacking before that date.

The relative risk was calculated as the observed number of breast cancer cases divided by the expected number. The expected number of breast cancers was obtained by multiplying the years at risk in 5 year age groups and calendar year periods by the breast cancer rate for the Danish female population. Ninety-five per cent confidence intervals were calculated using Byar's formula (Breslow and Day, 1987). Multiplicative Poisson regression models were used to investigate further possible risk factors such as calendar year, age and place of residence at the time of operation for breast hypertrophy, type of operation, surgical department, latency time (time since operation) and age at diagnosis of breast cancer. The Epicure program (Preston *et al.*, 1993) was used in the analyses.

### Results

In our study group of 1240 women operated on for breast hypertrophy, 32 cases of breast cancer were diagnosed, whereas 52.55 cases were expected if their risk was the same as that of the general population. This yielded a relative risk (RR) of 0.61 (95% CI 0.42-0.86) (see Table I).

We also looked at the risk of breast cancer on the basis of how much breast tissue was removed from the breast with the greatest reduction: <400 g, 400-600 g or >600 g. The 367 women with more than 600 g of tissue removed had a significantly lower risk than the general population (RR=0.30; 95% CI 0.10-0.69) at all time intervals after operation (latency). In addition, Table I shows how the RR varies with latency time. There appears to be a sharp drop in the RR 10-19 years after operation and an increase

Table I Relative risk of breast cancer for 1240 women with surgical reduction of breasts, operated on in Copenhagen, 1943–71, and followed until 1990 by latency and amount of breast tissue removed

| Time after operation (years) | Amount of breast tissue removed (g) | Number of women | Number of cases Obs | Exp | RR | 95% CI |
|---|---|---|---|---|---|---|
| All | Missing | 36 | 0 | 1.35 | – | – |
| | <400 | 468 | 15 | 18.65 | 0.80 | 0.45–1.33 |
| | 400–599 | 369 | 12 | 15.63 | 0.77 | 0.40–1.34 |
| | ⩾600 | 367 | 5 | 16.93 | 0.30 | 0.10–0.69 |
| | Total | 1240 | 32 | 52.55 | 0.61 | 0.42–0.86 |
| 0–9 | <400 | | 2 | 2.47 | 0.81 | 0.09–2.92 |
| | 400–599 | | 2 | 2.05 | 0.98 | 0.11–3.53 |
| | ⩾600 | | 1 | 2.46 | 0.41 | 0.01–2.27 |
| | Total | | 5 | 7.16 | 0.70 | 0.22–1.63 |
| 10–19 | <400 | | 2 | 5.30 | 0.38 | 0.04–1.36 |
| | 400–599 | | 0 | 4.08 | – | – |
| | ⩾600 | | 2 | 4.46 | 0.45 | 0.05–1.62 |
| | Total | | 4 | 14.24 | 0.28 | 0.08–0.72 |
| 20–29 | <400 | | 6 | 5.44 | 1.10 | 0.40–2.40 |
| | 400–599 | | 4 | 4.83 | 0.83 | 0.22–2.12 |
| | ⩾600 | | 1 | 5.28 | 0.19 | 0.00–1.05 |
| | Total | | 11 | 15.91 | 0.69 | 0.34–1.24 |
| >30 | <400 | | 5 | 5.44 | 0.92 | 0.30–2.14 |
| | 400–599 | | 6 | 4.67 | 1.29 | 0.47–2.80 |
| | ⩾600 | | 1 | 4.72 | 0.21 | 0.00–1.18 |
| | Total | | 12 | 15.23 | 0.79 | 0.41–1.38 |

Obs, observed; Exp, expected; RR, relative risk (Obs/Exp); 95% CI, 95% confidence interval.

afterwards. However, these results are highly dependent on the chosen latency time intervals, especially the limit between the first two intervals.

Table II shows the relative risks and confidence intervals stratified by age at the time of operation, corrected for the amount of tissue removed. The 36 women with an unknown amount of breast tissue removed were excluded in this analysis. Table II shows that the women aged 20 years or less at the time of the operation had a substantially (although not significantly) higher relative risk for breast cancer in comparison both with the general population and the other age groups. No significant variation in breast cancer risk was seen with type of operation, calendar year or place of operation, residence at the time of operation, or age at breast cancer diagnosis.

### Discussion

The risk for breast cancer decreases with the amount of breast tissue removed. This supports the hypothesis that potential foci for breast cancer are removed by the surgery. Table I shows that the risk is mainly reduced 10–19 years after the operation. A reduced risk would be expected in the first 10 years after the operation, if undiagnosed breast cancers were removed during the operation, and not 10–19 years later, as a breast cancer is considered to take at least 10 years to become clinically perceivable. An explanation for the increasing relative risk 20 years after the operation may be that the patients are still influenced by factors essential to the development of breast cancer.

The aetiology of breast hypertrophy for young women (<21 years) may differ from that for older women. Young women operated on for breast hypertrophy are often slim with large breasts, whereas the older women in general tend to be obese. This difference in aetiology could explain the increased risk among young women.

As breast cancer is usually located in the upper lateral quadrant of the breast, one might expect that surgical breast reduction in which tissue was removed from this area would lead to a further reduction in the breast cancer risk. However, as in most of the types of operations tissue is removed from the middle of the breast, our data are too few to investigate this hypothesis.

The hospital records did not contain consistent information on the distribution of known risk factors for breast cancer, such as high social class (Ewertz, 1988), nulliparity, high age at the time of first birth (Ewertz *et al.*, 1990; Adami

Table II Risk for breast cancer ($RR_1$) relative to the Danish female population by age at operation, and relative risk ($RR_2$) standardised for amount of breast tissue removed.

| Age at operation (years) | Number | Cumulative number | Obs | Exp | $RR_1$ | 95% CI | $RR_2$ | 95% CI |
|---|---|---|---|---|---|---|---|---|
| <21 | 132 | 132 | 4 | 2.23 | 1.79 | 0.48–4.59 | 2.78 | 0.85–9.03 |
| 21–30 | 434 | 566 | 9 | 13.15 | 0.68 | 0.31–1.30 | 1[a] | – |
| 31–40 | 292 | 858 | 11 | 15.00 | 0.73 | 0.37–1.31 | 1.07 | 0.44–2.57 |
| 41–50 | 239 | 1097 | 5 | 14.86 | 0.34 | 0.11–0.79 | 0.54 | 0.18–1.63 |
| >50 | 107 | 1204 | 3 | 7.28 | 0.41 | 0.08–1.20 | 0.70 | 0.19–2.60 |

$RR_1$, Obs/Exp; $RR_2$, relative risk in multiplicative Poisson model; [a]Reference category.

et al., 1990), early menarche, late menopause, adult height, hereditary characteristics (Adami et al., 1990) or hormone treatment (Adami et al., 1990; Colditz et al., 1995). We have no reason to believe, however, that the study population differs from the standard population in these respects.

The missing information on the 31 patients without hospital records and for the six women lost to follow-up does not seem to relate to breast cancer risk. At the first follow-up (1983), none of the 31 patients without hospital records had breast cancer.

Some of the women operated on for breast hypertrophy were probably obese, and as obese women have an increased risk for breast cancer (Adami et al., 1990) the study group should have a further decreased risk compared with obese women. Obese premenopausal women, however, have a decreased risk for breast cancer, possibly because of reduced ovarian activity (Adami et al., 1990).

No studies evaluating the risk of breast cancer for women with breast hypertrophy could be found, but Hsieh and Trichopoulos (1991) found that the size of the breast is a risk factor for breast cancer for post-menopausal women, even after correction for obesity. As our study group is characterised by large breasts, the relative risk would have been further reduced compared with women with large breasts without reduction mammoplasty. Our study population contains a preponderance of women from Copenhagen and its suburbs, where there is a slightly higher breast cancer risk than in the rest of Denmark. Hence, the expected number of cancer cases was too low (up to 15%), compared with Copenhagen women.

In conclusion, the women in this study experienced a lower risk of breast cancer than the Danish female population. The study therefore does not support the hypothesis that damage to the mammary ducts due to the operation increases the risk for cancer. On the contrary, this study indicates that reduction mammoplasty decreases the incidence of breast cancer and that the decrease is larger the more breast tissue removed.

**Acknowledgements**
Many thanks to Marianne Ewertz and Geert Schou, who assisted us with invaluable advice.

### References

ADAMI H-O, ADAMS G, BOYLE P, EWERTZ M, LEE NC, LUND E, MILLER AB, OLSSON H, STEEL M, TRICHOPOULOS D AND TULINIUS R. (1990). Breast-cancer etiology. *Int. J. Cancer*, Suppl 5, 22–39.

BRESLOW NE AND DAY NE. (eds) (1987). *Statistical Methods in Cancer Research. Vol II. The Design and Analysis of Cohort Studies*. IARC Scientific Publication No. 82. pp. 69–71. International Agency for Research on Cancer: Lyon.

COLDITZ GA, HANKINSON SE, HUNTER DJ, WILLETT WC, MANSON JE, STAMPFER MJ, HENNEKENS C, ROSNER B AND SPEIZER FE. (1995). The use of estrogens and progestins and the risk of breast cancer in postmenopausal women. *N. Engl. J. Med.*, **332,** 1589–1593.

EWERTZ M. (1988). Risk of breast cancer in relation to social factors in Denmark. *Acta Oncol.*, **27,** 787–793.

EWERTZ M, DUFFY SW, ADAMI H, KVÅLE G, LUND E, MEIRIK O, MELLEMGAARD A, SOINI I AND TULINIUS H. (1990). Age at first birth, parity, and risk of breast cancer: A meta-analysis of 8 studies from the Nordic countries. *Int. J. Cancer*, **46,** 597–603.

FEKETE E AND GREEN CV. (1936). The influence of complete blockage of the nipple on the incidence and location of spontaneous mammary tumors in mice. *Am. J. Cancer*, **27,** 513–515.

HSIEH C-C AND TRICHOPOULOS D. (1991). Breast size, handedness and breast cancer risk. *Eur. J. Cancer*, **27,** 131–135.

LUND K, EWERTZ M AND SCHOU G. (1987). Breast cancer incidence subsequent to surgical reduction of the female breast. *Scand. J. Plast. Reconstr. Surg.*, **21,** 209–212.

PRESTON DL, LUBIN JH, PIERCE DA AND MCCONNEY ME. (1993). *Epicure*. Hirosoft International: Seattle.

REES TD AND COBURN R. (1972). Breast reduction: Is it an aid to cancer detection? *Br. J. Plast. Surg.*, **25,** 144.

STRÖMBECK JO. (1964). Macromastia in women and its surgical treatment. *Acta Chir. Scand.*, Suppl 341, 111–112.


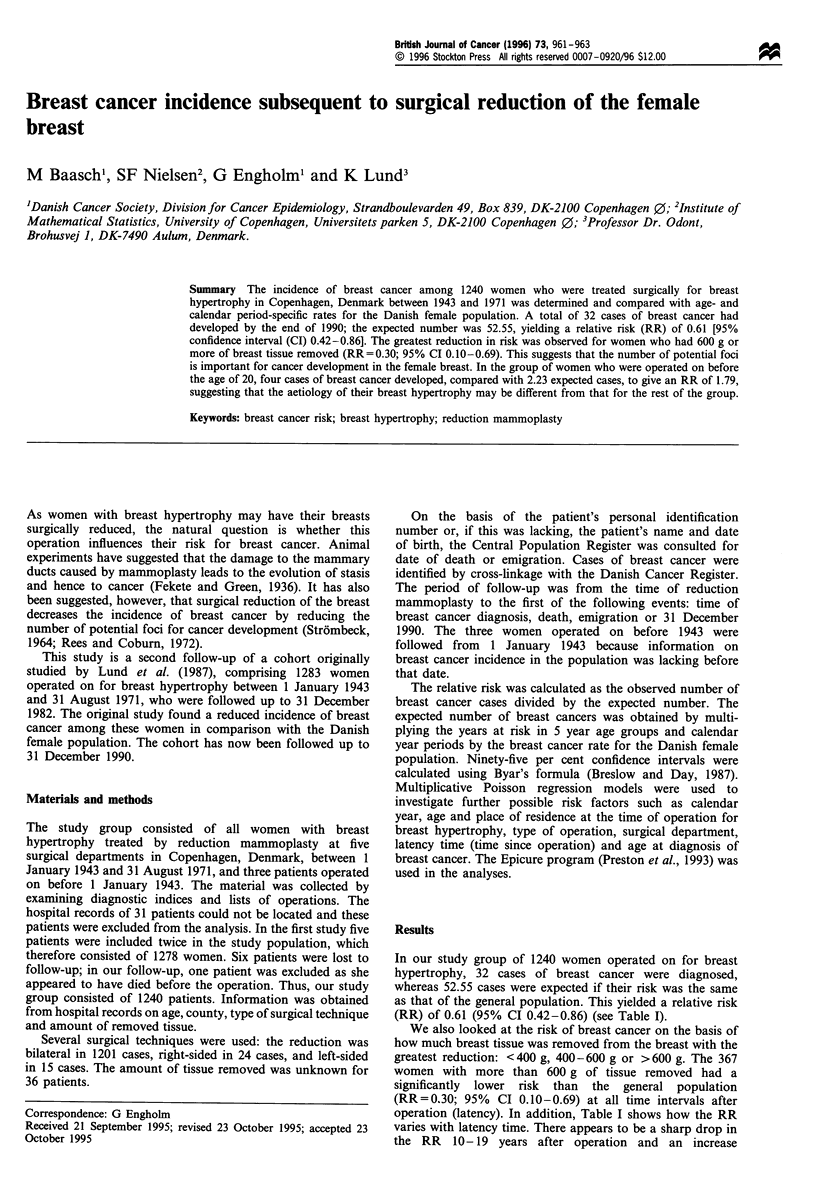

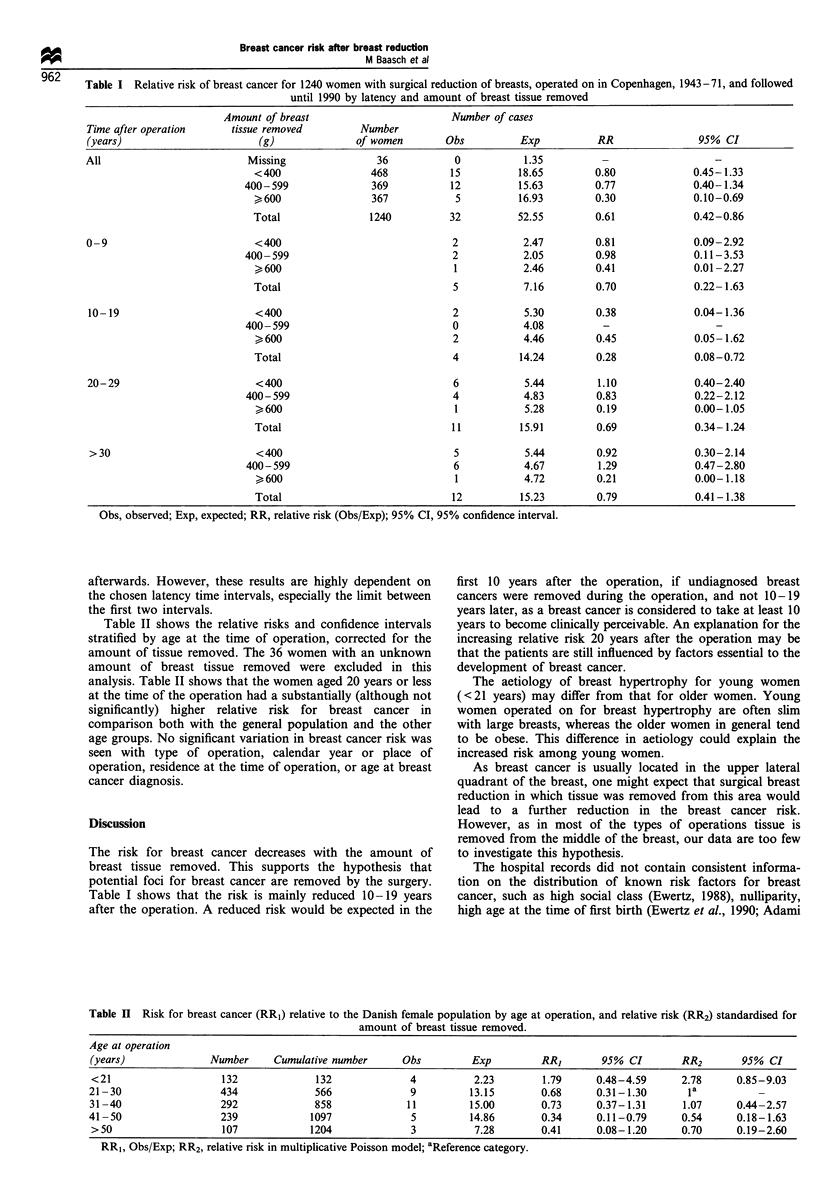

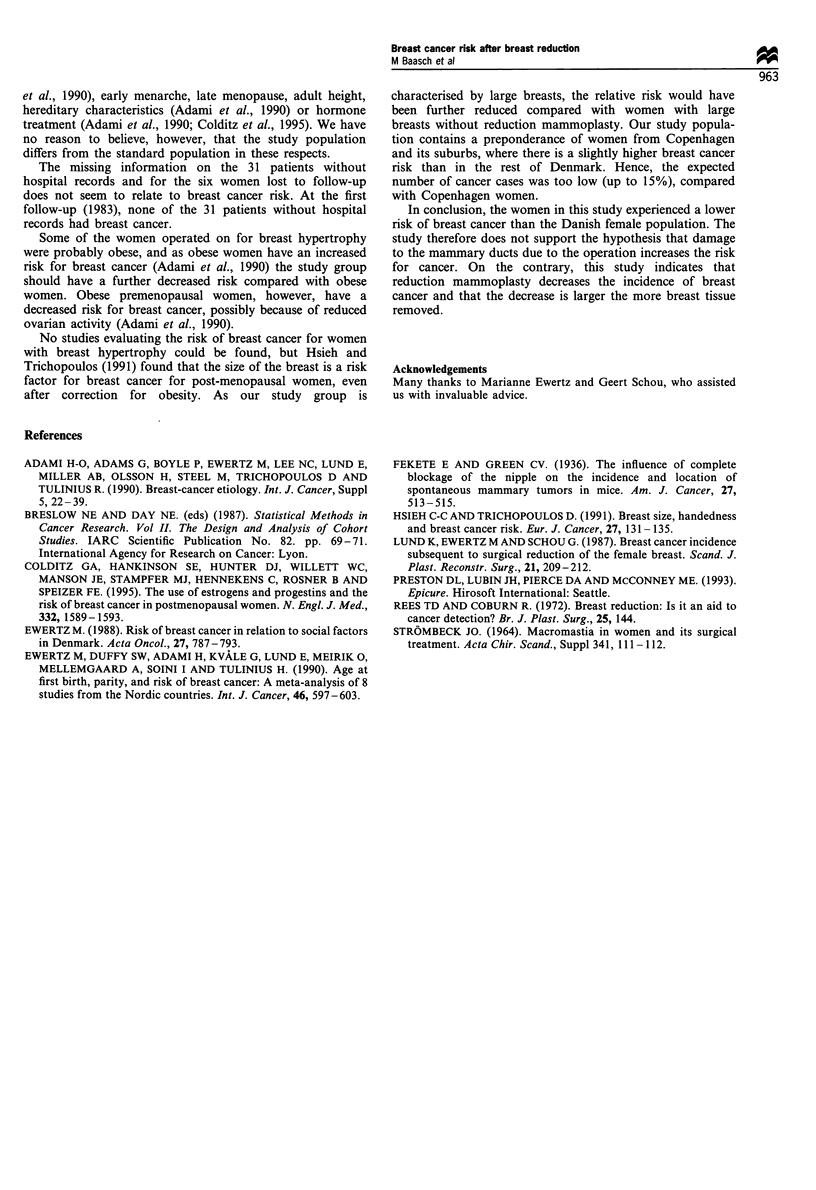


## References

[OCR_00296] Adami H. O., Adams G., Boyle P., Ewertz M., Lee N. C., Lund E., Miller A. B., Olsson H., Steel M., Trichopoulos D. (1990). Breast-cancer etiology. Report of a working party for the Nordic Cancer Union.. Int J Cancer Suppl.

[OCR_00308] Colditz G. A., Hankinson S. E., Hunter D. J., Willett W. C., Manson J. E., Stampfer M. J., Hennekens C., Rosner B., Speizer F. E. (1995). The use of estrogens and progestins and the risk of breast cancer in postmenopausal women.. N Engl J Med.

[OCR_00316] Ewertz M., Duffy S. W., Adami H. O., Kvåle G., Lund E., Meirik O., Mellemgaard A., Soini I., Tulinius H. (1990). Age at first birth, parity and risk of breast cancer: a meta-analysis of 8 studies from the Nordic countries.. Int J Cancer.

[OCR_00312] Ewertz M. (1988). Risk of breast cancer in relation to social factors in Denmark.. Acta Oncol.

[OCR_00330] Hsieh C. C., Trichopoulos D. (1991). Breast size, handedness and breast cancer risk.. Eur J Cancer.

[OCR_00334] Lund K., Ewertz M., Schou G. (1987). Breast cancer incidence subsequent to surgical reduction of the female breast.. Scand J Plast Reconstr Surg Hand Surg.

[OCR_00341] Rees T. D., Coburn R. (1972). Breast reduction: is it an aid to cancer detection?. Br J Plast Surg.

[OCR_00345] STROEMBECK J. O. (1964). MACROMASTIA IN WOMEN AND ITS SURGICAL TREATMENT. A CLINICAL STUDY BASED ON 1,042 CASES.. Acta Chir Scand Suppl.

